# Machine learning-based technique for resonance and directivity prediction of UMTS LTE band quasi Yagi antenna

**DOI:** 10.1016/j.heliyon.2023.e19548

**Published:** 2023-09-01

**Authors:** Md. Ashraful Haque, Dipon Saha, Samir Salem Al-Bawri, Liton Chandra Paul, Md Afzalur Rahman, Faisal Alshanketi, Ali Alhazmi, Ali Hanafiah Rambe, M.A. Zakariya, Saeed S. Ba Hashwan

**Affiliations:** a Department of Electrical and Electronic Engineering, Universiti Teknologi PETRONAS, Bandar Seri Iskandar, 32610, Perak, Malaysia; b Department of Electrical and Electronic Engineering, Daffodil International University, Dhaka, 1341, Bangladesh; c Space Science Centre, Climate Change Institute, Universiti Kebangsaan Malaysia (UKM), 43600, Bangi, Malaysia; d Department of Electronics & Communication Engineering, Faculty of Engineering & Petroleum, Hadhramout University, Al-Mukalla, 50512, Hadhramout, Yemen; e Department of Electrical, Electronic and Communication Engineering, Pabna University of Science and Technology, Pabna, Bangladesh; f Department of Computer Science, Jazan University, Jazan, Saudi Arabia; g Department of Information Technology and Security, Jazan University, Jazan, 45142, Saudi Arabia; h Department of Electrical Engineering, Universitas Sumatera Utara, Medan, Indonesia

**Keywords:** Quasi Yagi-Uda, UMTS 2100 MHz, LTE, CST, ADS, Machine learning

## Abstract

In this study, we have presented our findings on the deployment of a machine learning (ML) technique to enhance the performance of LTE applications employing quasi-Yagi-Uda antennas at 2100 MHz UMTS band. A number of techniques, including simulation, measurement, and a model of an RLC-equivalent circuit, are discussed in this article as ways to assess an antenna's suitability for the intended applications. The CST simulation gives the suggested antenna a reflection coefficient of -38.40 dB at 2.1 GHz and a bandwidth of 357 MHz (1.95 GHz-2.31 GHz) at a -10 dB level. With a dimension of 0.535$\lambda_{0}\times$0.714$\lambda_{0}$, it is not only compact but also features a maximum gain of 6.9 dB, a maximum directivity of 7.67, VSWR of 1.001 at center frequency and a maximum efficiency of 89.9%. The antenna is made of a low-cost substrate, FR4. The RLC circuit, sometimes referred to as the lumped element model, exhibits characteristics that are sufficiently similar to those of the proposed Yagi antenna. We use yet another supervised regression machine learning (ML) technique to create an exact forecast of the antenna's frequency and directivity. The performance of machine learning (ML) models can be evaluated using a variety of metrics, including the variance score, R square, mean square error (MSE), mean absolute error (MAE), root mean square error (RMSE), and mean squared logarithmic error (MSLE). Out of the seven ML models, the linear regression (LR) model has the lowest error and maximum accuracy when predicting directivity, whereas the ridge regression (RR) model performs the best when predicting frequency. The proposed antenna is a strong candidate for the intended UMTS LTE applications, as shown by the modeling results from CST and ADS, as well as the measured and forecasted outcomes from machine learning techniques.

## 1 Introduction

The technology behind wireless cellular networks has made significant advances in recent years, which has paved the path for the development of a wide variety of innovative applications that go well beyond simple phone conversations [1]. With each new generation of technology that has been developed, mobile devices have seen improvements in the amount of data that can be sent, the quality of their connections, and the functionality of their applications [2]. Long Term Evolution (LTE), the most recent generation of mobile communication technology, is being implemented in a considerable number of mobile devices, such as smartphones, laptops, and tablets [3]. This is due to the fact that LTE is capable of providing high spectrum efficiency, high-speed transmission, and high data rate capabilities. LTE makes use of a frequency range for its operations that begins at 400 MHz and goes all the way up to 4 GHz [4]. Yagi-Uda antennas have been implemented into a range of unidirectional designs [5], [6] in order to meet the needs of LTE applications. Mobile radio communications have evolved from analog systems in the late 80s, which could carry only voice. To more robust GSM/GPRS voice systems, enabling the boom in text messaging to the latest advances in mobile broadband brought by UMTS, HSDPA, and LTE [7]. LTE networks employ from 700 MHz to 2300 MHz bands, while UMTS networks mostly use the 1.9 GHz and 2.1 GHz bands [7]. The Universal Mobile Telecommunications System [8] is one of the third-generation (3G) cell phone technologies that is also being developed into a fourth-generation (4G) technology. The study recommended a Yagi-shaped microstrip antenna for use in the LTE communication system at the UMTS 2100 MHz frequency. In order to construct and fine-tune the antenna, this research employs CST MWS simulation software.

Table 1 shows a comparison of many ongoing parallel activities. Reference papers [9], [10], [11], [12], [13], [14] report reflection coefficients of -35.58 dB, -27 dB, -25.02 dB, -35 dB, -29 dB, and -32 dB, respectively; nevertheless, in the suggested Yagi antenna, it is observed as -38.4 dB in CST. According to CST's calculations, the proposed design has a peak gain of 6.9 dBi, as opposed to the 5.6 dBi, 6.46 dBi, 6.73 dBi, 5.02 dBi, 4.27 dBi, 4.3 dBi, and 5 dBi reported by the references. Reference articles [9], [10], [12], [13], [14] report radiation efficiencies of 82.4%, 70%, 80%, 73%, and 96%, respectively; however, 89.9% is measured in CST for the proposed Yagi antenna. The investigations based on machine learning are not employed in the references that have been listed above; even so, they are extensively used in the design that has been presented. Moreover, the RLC equivalent circuit has also been implemented in the prosed Yagi antenna, which is not described in the literature that was previously referred to. The merits of using a Yagi antenna are typically between 50 and 70 degrees. Strong signals improve mobile phone communication. Gain can also be increased by combining several omnidirectional antennas with a VHF Yagi Antenna. Mobile phone signals may be strengthened and enhanced by installing a Yagi antenna. One must accept the drawbacks of using a long Yagi antenna to get a high gain level. A High Gain Yagi Antenna must combine several components and have additional directors to use these antennas. A long antenna has its advantages, but it also has its drawbacks. In the previous research works, the implementation of an equivalent circuit under the transmission line is presented by a parallel circuit using only the value of inductance (L) and capacitance (C) [15]. An equivalent circuit is used to describe the filter layout and antenna configuration completely. It has been reported that the location of the central resonant frequency at return levels below -20 dB has shifted due to variations in L-C values. It is crucial to design the antenna parameters, thus achieving a return loss level below -10 dB [16]. In order to solve this issue, this research added the value of resistance in the equivalent circuit in parallel with the L-C value for achieving a return loss level below -10 dB. As such, the proposed comparable circuit analysis ignores how the resistance value affects the return loss. At last, the R, L, and C values for every model can be calculated from the location of their resonant frequency.

**Table 1:** Performance comparisons with the published state of the art.

Parameter	Ref. [9]	Ref. [20]	Ref. [10]	Ref. [11]	Ref. [12]	Ref. [13]	Ref. [14]	Proposed
Method	Tri band Rectenna	Quasi Yagi antenna	Split-ring resonator	Slotted array antenna	Dielectric resonator antenna	Quasi Yagi (2 port)	Finite integration technique	Single port Quasi Yagi Antenna
Return loss (dB)	-35.58	—	-27	-25.02	-35	-29	-32	-38.40
Operating Frequency (GHz)	0.9, 1.83, 2.11	6, 8	1.8, 2.45	28	3.7	3.6	3.8, 5.2	2.1
Bandwidth (GHz)	0.94 -2.08	5.03-9.39	1.7-1.9 2.39-2.55	27.03-28.82	3.3-4.2	3.48-3.8	2.2-8	1.95-2.31
Peak Gain (dBi)	5.6	6.46	6.73	5.02	4.27	4.3	5	6.9
Radiation Efficiency %	82.4	—	70	—	80	73	96	89.9
Size (W×L)	0.336*λ*_0_× 0.264 *λ*_0_	0.545*λ*_0_× 0.55*λ*_0_	0.593*λ*_0_× 0.48*λ*_0_	13.63*λ*_0_× 6.36*λ*_0_	0.29*λ*_0_× 0.19*λ*_0_	0.47*λ*_0_× 0.93*λ*_0_	0.7*λ*_0_× 0.467*λ*_0_	0.535*λ*_0_× 0.714*λ*_0_
ML investigations	No	No	No	No	No	No	No	Yes
RLC Equivalent Circuit	No	No	No	No	No	No	No	Yes
Substrate Material	FR4	TRF-45	FR4	AD430	FR4	FR4	FR4	FR4

However, getting an antenna to function well enough is difficult and time-consuming, utilizing 3D electromagnetic simulation tools like CST, HFSS, FEKO, and ADS. Many academics have started using machine learning (ML) strategies to optimize antenna design, predict results, and select antennas for wireless applications [17], [18]. This allows them to function in spite of the aforementioned constraints. Several ML methods [19] have been developed to predict antenna outcomes such as resonance frequency ($f_{r}$), gain, return loss (S11), bandwidth, and so on. Antenna performance is optimized with the use of CST MWS simulation software in this article. In addition, the same antenna is redesigned in measurement to verify the performance result achieved in the simulation. The Advance Design System (ADS) circuit simulation tool is used to verify the return loss and bandwidth using the R-L-C equivalent circuit. This paper is unique in that it combines the integration of simulation, measurement, and construction of the RLC equivalent circuit model with a comparison of the CST result with Agilent ADS, as well as the use of multiple regression models to evaluate the performance and errors of the proposed antenna. This is the article's innovative contribution.

## 2 Design of proposed antenna

**Figure 1:**
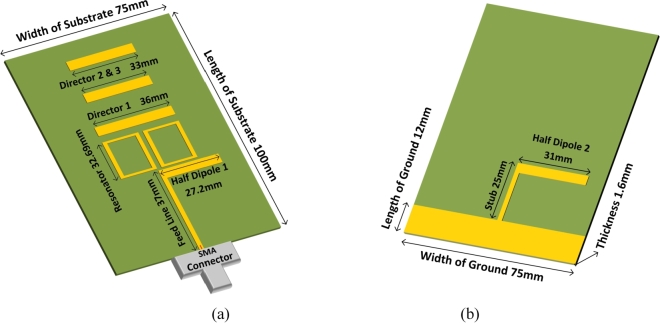
(**a**) Geometry of quasi-Yagi antenna (Front). (**b**) Geometry of quasi-Yagi antenna (Back).

The Quasi-Yagi antenna is designed and simulated with the help of the CST (Computer Simulation Technology) program. A copper metal ground plane is employed as part of the antenna's construction together with the driving element, directors, box resonator elements, and ground plane. Copper metal and the substrate are 0.035 mm and 1.6 mm thick, respectively; the dielectric constant of FR-4 is 4.3, and the loss tangent is 0.025. Fig. 1 includes all the available dimensions of the following elements. Fig. 1(a) depicts the geometry behind the quasi-Yagi antenna (Front) whereas Fig. 1(b) explains the geometry behind a quasi-Yagi antenna (Back). The dimensions of the substrates are as follows: width (Ws) = 75 mm, length (Wl) = 100 mm, ground (Lg) = 12 mm, feedline is 37 mm and thickness (Ts) = 1.6 mm. Director 1 of the Yagi antenna is 36 mm in diameter, whereas Directors 2 and 3 are each 33 mm.

## 3 Working principle

The simulated current distribution is analyzed and explained to provide light on the attributes and operating principles of the proposed antenna.

### 3.1 Current distribution

**Figure 2:**
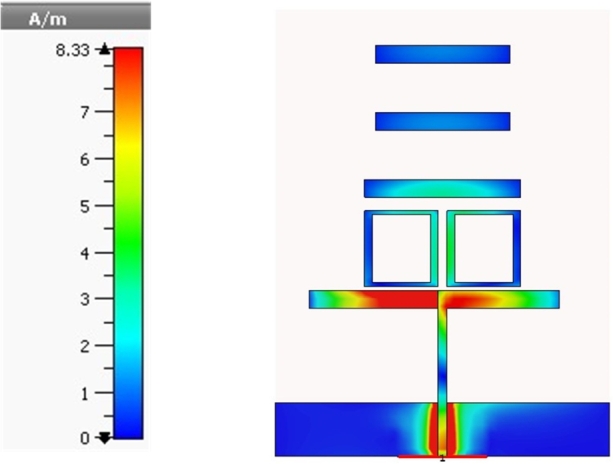
Simulated current distribution of the proposed antenna at 2100 MHz.

The Yagi antenna's surface current distribution at 2.1 GHz is displayed in Fig. 2 to be at its highest point (32.87 A/m) at the lower part of the feed line and the lower borders of the patch. The color is a visual representation of the density of the surface current. At 2.1 GHz, there is a detectable current running down the surface of the object. However, the surface current has a greater degree of concentration and intensity on a reversed version of the L form.

## 4 Parametric study

For gaining a further understanding of the structure, subsequent sections demonstrate the impact of its key parameters.

### 4.1 Director of Yagi antenna

**Figure 3:**
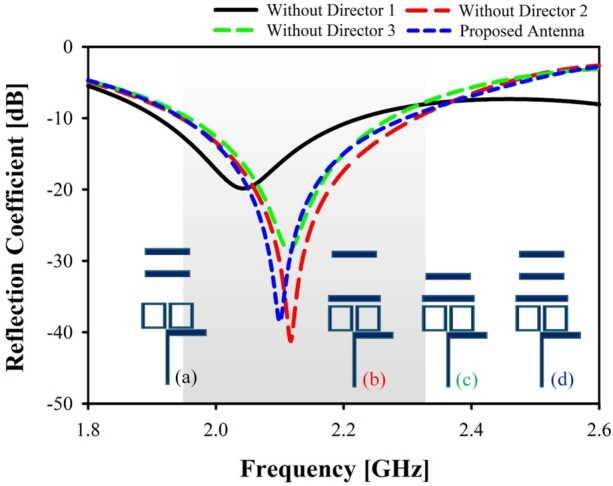
Simulated reflection coefficient for different director: (**a**) without director 1, (**b**) without director 2, (**c**) without director 3, (**d**) with all directors (proposed antenna).

**Figure 4:**
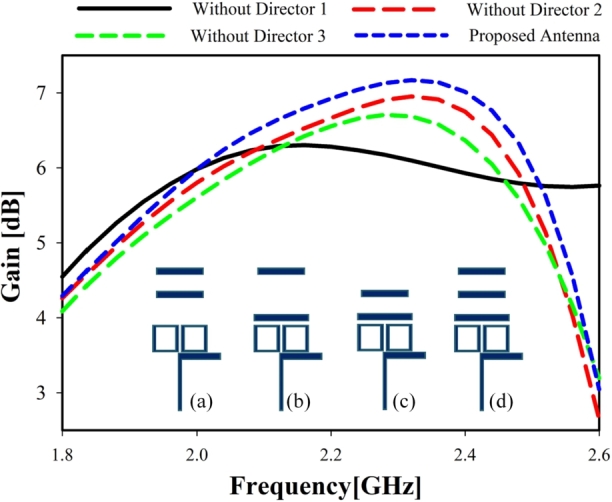
Simulated gain for different directors: (**a**) without director 1, (**b**) without director 2, (**c**) without director 3, (**d**) with all directors (proposed antenna).

It is common knowledge that the gain of a Yagi antenna is proportional to its length since more directors can boost the antenna's end-fire performance [21]. Fig. 3 shows the proposed antenna operating in a single band without and with the directors. The antenna has the best S11 curve when employing all three directors simultaneously. The intended antenna with all three directors present is shown in blue, whereas the absence of director 1 is shown in black, director 2 in red, and director 3 in green. Furthermore, the number of directors added to the antenna after the dipole determines how much gain the antenna will have [22]. Fig. 4 illustrates the proposed antenna without and with directors. Directors 2 and 3 can gain more than 6 dB, but director 1 can gain less than 6 dB. Maximum gain is 7 dB with all three directors.

### 4.2 Box resonator of proposed antenna

**Figure 5:**
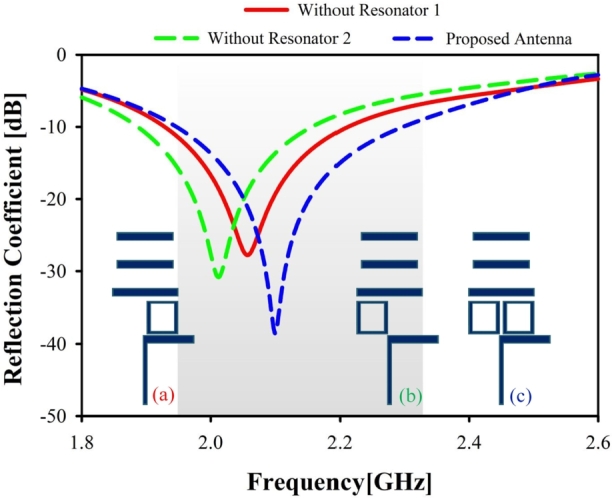
Simulated reflection coefficient for different resonator: (**a**) without resonator 1, (**b**) without resonator 2, (**c**) with 2 resonators (proposed antenna).

There has been a lot of focus on resonator structure to reach high bandwidth [23]. The suggested antenna is shown in operation in the LTE band in Fig. 5 both without and with the resonator. The return loss was found to rise once the two-box resonator was included. Using all box resonators at once yields the optimal S11 curve for the antenna. The proposed antenna is shown in blue with both box resonators present, while the red and green colors stand for the absence of box resonators 1 and 2, respectively.

## 5 Result and discussion

**Figure 6:**
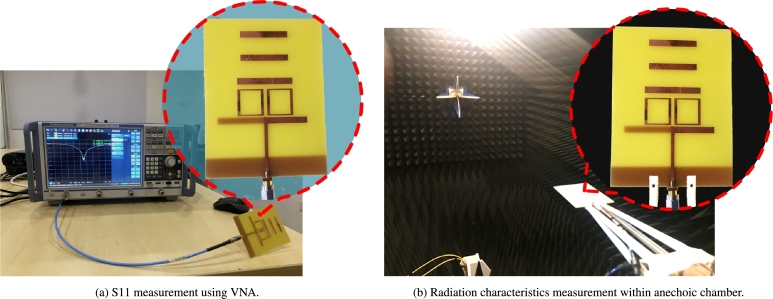
Measurement setup.

**Figure 7:**
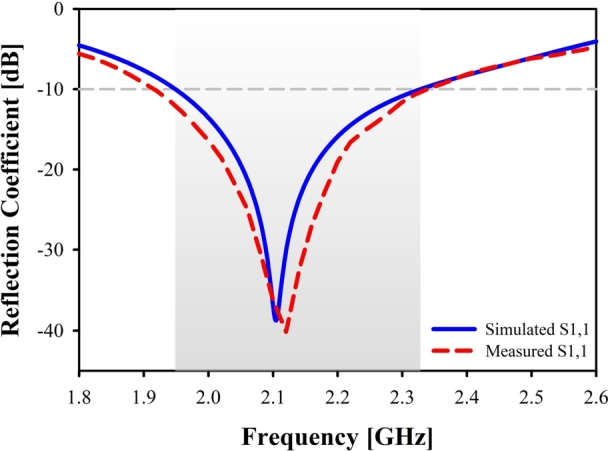
Simulated and Measured reflection coefficient of the proposed antenna.

Power reflections within a transmission line are quantified by the return loss (S11). The term “return loss” is used to describe how weak a signal is as it is reflected off an antenna and sent back to the source (S1,1) [24]. As can be seen in Fig. 6(a), fabricated prototype and a vector network analyzer (VNA) is utilized in order to test the port qualities, whereas an anechoic chamber is utilized in Fig. 6(b), in order to evaluate the radiation properties. It is clear that the resonant frequency that was simulated and the one that was measured is very near to one another (Simulated: 2.10 GHz and Measured: 2.12 GHz). At the resonant point, the reflection coefficient has a value of approximately -38.40 dB (when simulated) and -40.14 dB (when measured) in Fig. 7. The measured result is in good agreement with the simulated findings over the UMTS band, with only minimal misalignment and harmonics.

**Figure 8:**
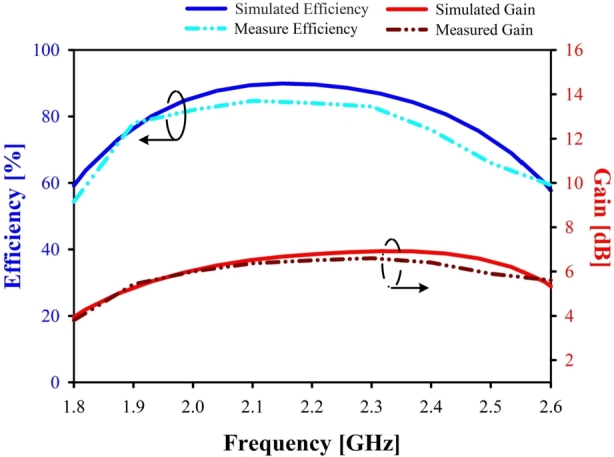
Simulated and Measured Gain & efficiency of the proposed antenna.

**Figure 9:**
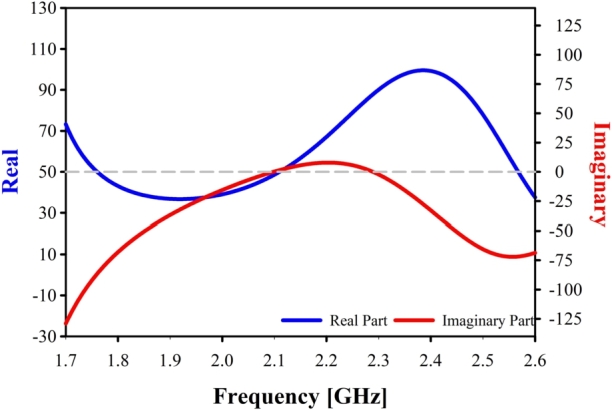
Z-parameter of the studied antenna.

When evaluating an antenna's performance, it is crucial to evaluate both the antenna's gain and its directivity. The term “gain” is used to describe the amount of energy that is transferred to the primary beam, while the term “directivity” describes the amount of energy that is concentrated in a single direction [25]. Using an antenna's Gain and Directivity values, one may determine the antenna's efficiency [26]. As shown in Fig. 8, the proposed antenna has a maximum gain of 6.9 dB and a maximum efficiency of 89.9% at the band of interest. Fig. 9 highlights another critical aspect of the proposed Yagi antenna: the Z-matrix. This figure states that the real part of the Z-parameter is close to 50, while the imaginary part of the Z-parameter is close to 0 at 2.10 GHz. Having a low impedance confirms that the proposed antenna is very near to being a pure resistive device, which is a good sign of impedance matching.

**Figure 10:**
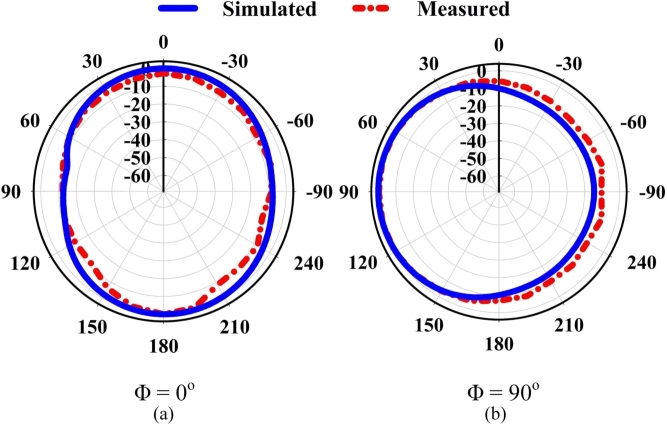
Simulated and measured 2D rad. patterns for the Yagi antenna for Φ = 0^∘^, 90^∘^.

Fig. 10 displays the 2D radiation patterns measured and simulated at 2.1 GHz. For example, Fig. 10(a) depicts the XZ cut (Φ = 0^∘^), also known as the E-plane of the radiation pattern, occurs when theta and phi are spherical coordinates aligned to the Cartesian axes. In contrast side, the configuration, which is equal to a constant 90 degrees and then ranges from 0 to 360 degrees, is referred to Fig. 10(b), as the YZ cut (Φ = 90^∘^) and it is also referred to as the H-plane. The simulated two-dimensional radiation patterns of the Yagi antenna are carried out into the E-plane at yz direction and xz direction within phi = (90^∘^, 0^∘^). The lobe's primary focus corresponding to 2.1 GHz is located at (177^∘^, 90^∘^) for (0^∘^, 90^∘^). At angles of 0 and 90 degrees, the amplitude of the primary lobe of the E-field is 11.2 and 21.4 dBV/m, respectively. Radiation in all directions has been tested, and the recommended prototype has been found to operate satisfactorily in simulations. However, due to limitations of the measurement setup and faults in the 3D Yagi antenna, a slight discrepancy is investigated between the simulated and measured results in both planes.

### 5.1 Equivalent circuit model

The equivalent circuit of the antenna is derived from impedance analysis tools of the designed antenna using CST Studio simulation and circuit design tools in Agilent ADS software. Maximum power transfer (at least 90%) from the input port to the antenna structure and radiation into free space is guaranteed by a return level of less than -10 dB at the resonance frequency. When the impedance of the antenna circuit is matched to the characteristic impedance of 50 Ω, the maximum amount of power can be transferred [27]. According to the principle of maximum power transmission, for a network to be considered “matched,” the load impedance and the input resistance (Z${}_{load}$ = R${}_{in}$) should be as close to equal as possible [27].

**Figure 11:**
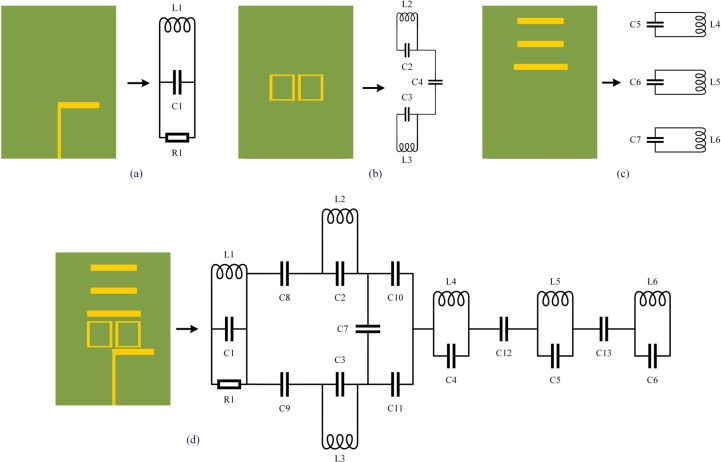
Evolution of equivalent circuit of Yagi antenna: (**a**) Circuit model for dipole element, (**b**) Circuit model for box resonator, (**c**) Circuit model for three directors, (**d**) Final obtained equivalent circuit model.

**Figure 12:**
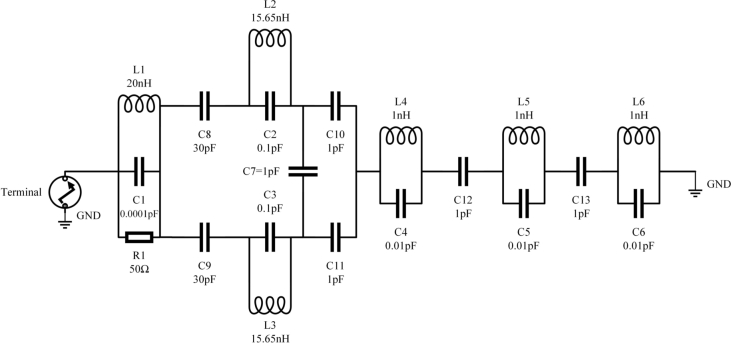
Final approximated equivalent circuit model after adjusting resistance, capacitance and inductance values.

**Figure 13:**
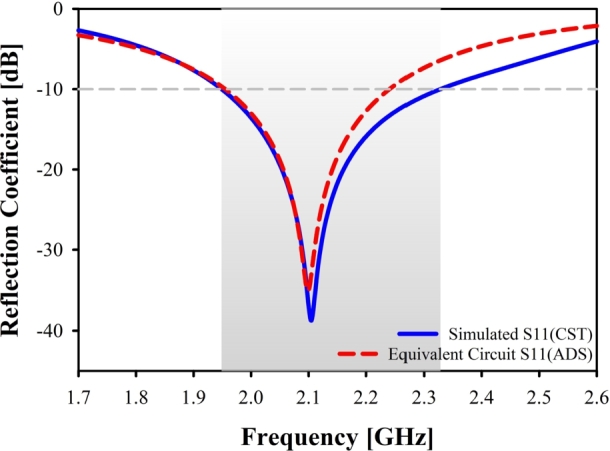
Simulated reflection coefficient of equivalent circuit in ADS and CST.

The approach relies on locating a lumped element model (RLC circuit) with close enough characteristics to the proposed Yagi antenna to be helpful. An equivalent circuit is suggested for each component of the designed antenna, as depicted in Fig. 11 (a), (b), (c), and the antenna is then reassembled in Fig. 11 (d). [28], [29]. In the final step, a simulation is performed using the R L C parameters to cover the complete frequency range using the equivalent circuit model of the proposed antenna, as shown in Fig. 12. The RLC equivalent result is in good agreement with the findings obtained through simulation across the UMTS spectrum, with just a small amount of mismatch occurring. This model reproduces, to a close approximation, the behavior of the proposed Yagi antenna. The properties of the notches were very apparent, as seen in Fig. 13.

#### 5.1.1 Equivalent circuits of Yagi antenna dipole element

As part of the equivalent circuit, the proposed Yagi antenna was developed using transmission lines as depicted in Fig. 11 (a) Consequently, a dipole element of the antenna reproduces a parallel R1, L1, C1 circuit. C8 represents the gap between the dipole and box resonator 1, whereas C9 represents the gap between the dipole and box resonator 2.

#### 5.1.2 Equivalent circuits of box resonator

In Fig. 11 (b), the first box resonator is denoted by the values C2 and L2, while the second box resonator is denoted by the parameters C3 and L3, and the gap between the two box resonators is denoted by C7. The gap between box resonators and director 1 is represented by C10 and C11, respectively.

#### 5.1.3 Equivalent circuits of directors

The combination C4 and L4 symbolizes the first director, C5 and L5 denotes the second director, and C6 and L6 indicate the third director. The gap between director 1 and director 2 is denoted by the symbol C12, while the gap between director 2 and director 3 is signified by the letter C13 as shown in Fig. 11 (c).

### 5.2 Machine learning based techniques

#### 5.2.1 Methodology of machine learning

The methodology consists of two distinct sections. The first stage of the procedure involves utilizing the CST simulation software to construct the antenna designed for operation within the UMTS 2100 MHz frequency range in the context of the LTE application. Additionally, the software is employed to extract the dataset generated utilizing a parametric sweep. The subsequent phase involves training the dataset in order to implement machine learning models and predict the most effective model.

**Figure 14:**
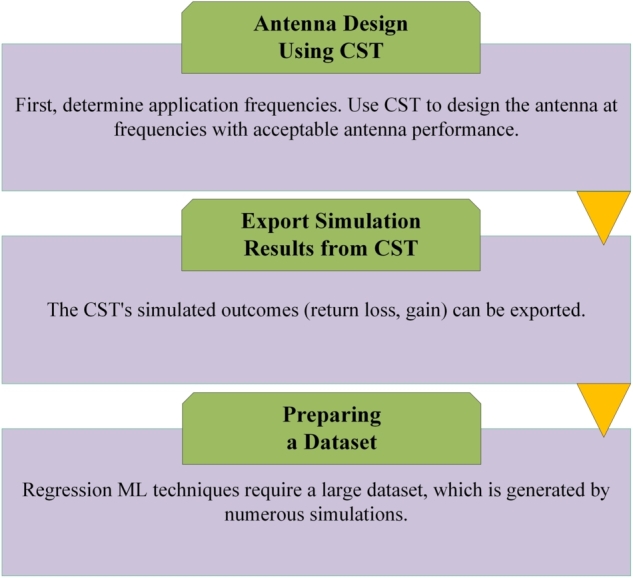
Data acquisition workflow for Machine Learning.

The methodology depicted in Fig. 14 is going to be expounded upon. Initially, it is imperative to ascertain the frequency at which the UMTS 2100 MHz LTE application operates. The application from Computational Electromagnetics (CEM) software, specifically CST (Computer Simulation Technology), is employed to facilitate the design of an antenna that operates at frequencies where its performance is deemed satisfactory. By employing a parametric sweep technique, extracting the simulated parameters of CST, including the director's length, dipole size, ground length, and reflector dimensions, is feasible. In certain instances, regression machine learning algorithms may benefit from utilizing larger datasets; however, it is important to note that this is not universally applicable. Various factors, such as the complexity of the problem, the dimensionality of the input characteristics, and the model's complexity, influence a larger dataset's impact on a regression model. Ultimately, a total of 75 data samples are obtained through the implementation of CST MWS simulation software. Subsequently, a range of regression machine learning (ML) techniques is employed to forecast the gain and resonant frequency of the proposed Yagi antenna. The dataset can be partitioned into distinct subsets for training and testing purposes by employing the train-test split technique. In this approach, the dataset is randomly divided into two distinct categories: one for training the model and the other for evaluating its performance by assessing its accuracy on previously unseen data.

**Figure 15:**
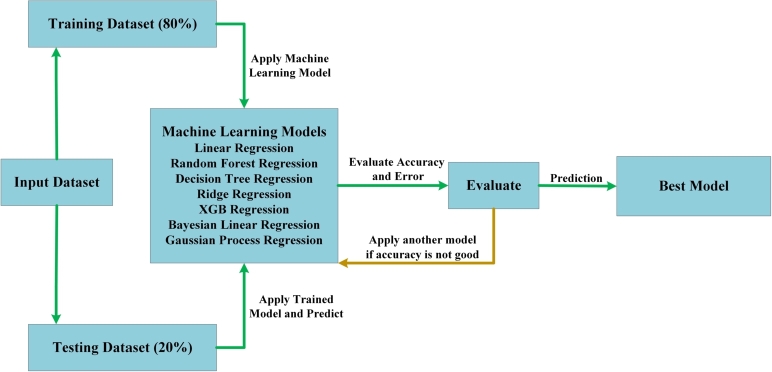
Data acquisition workflow for Machine Learning.

The current study utilizes seven different algorithms based on machine learning for the purpose of generating predictions. The regression models being considered encompass Linear Regression, Random Forest Regression, Decision Tree Regression, Ridge Regression, XGB Regression, Bayesian Linear Regression, and Gaussian Process Regression. The selection of these algorithms is predicated upon their capacity to conduct regression analysis on datasets exhibiting non-linear characteristics. The utilization of regression is deemed the most appropriate methodology for making predictions, given that the desired result involves numerical values. An error, which is a fundamental statistic in regression analysis, is given its name due to its widespread occurrence. The flowchart presented in Fig. 15 provides a visual representation of the sequential steps involved in the development process of a machine learning algorithm. After conducting a parametric sweep using the CST simulation software, the dataset was analyzed and subsequently divided into two distinct segments. The research pertaining to machine learning was carried out exclusively within the simulated Python environment provided by Google, known as Google Colab. The sci-kit learn machine learning framework was utilized for the efficient construction of the Regression models. The utilization of Matplotlib was observed in all analyses and visualizations, with particular emphasis on its application in conclusion.

In accordance with the recommendation proposed in [30], the initial phase of the study entailed the selection of 80% of the complete dataset for the purpose of training. At the same time, the remaining 20% was allocated for testing in the subsequent phase. Afterward, the training dataset is exposed to a machine-learning algorithm that integrates different features and labels. After the completion of model training and cross-validation, the model can be efficiently employed to predict the resonant frequency and realized gain for the specified inputs. The utilization of machine learning (ML) facilitates the generation of faster and more accurate predictions compared to the outcomes achieved through computer simulation technology (CST). According to the forecast, the Ridge Regression model is deemed optimal for predicting resonant frequency, while the Linear Regression model is considered optimal for predicting directivity.

#### 5.2.2 Machine learning models

**Figure 16:**
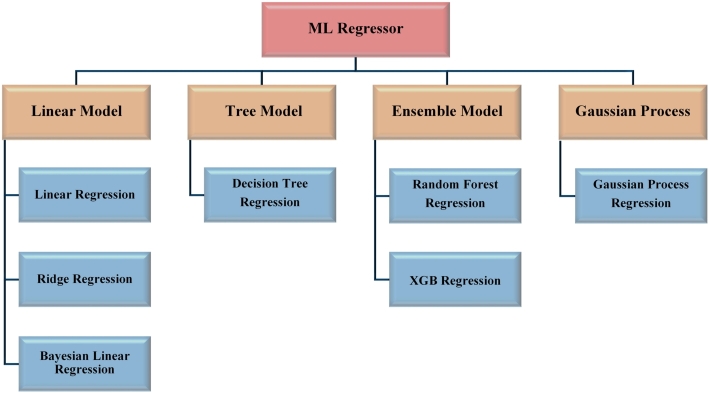
Tabulation of the Machine Learning models.

When it comes to achieving optimal performance, having access to a wide variety of models is invaluable. Cause-and-effect relationships between variables can be evaluated using regression analysis [31], a statistical technique. We use regression analysis because it solves our problem. So, we employed eight of the most useful machine learning regression models as depicted in Fig. 16. Following is a condensed explanation of each of these.

**Decision Tree Regression:** Regression trees predict continuous target variables like numbers [32]. Decision tree regression predicts continuous target variables using supervised machine learning. It is a decision tree algorithm variant for classification tasks.

**Linear Regression:** Linear regression [33] maintains a linear connection between independent and dependent variables. Thus, independent variables alter correspondingly to dependent factors. Errors (the variations between expected and actual values) are normally distributed and have constant variance, another crucial assumption.

**Ridge Regression:** When working with a large number of parameters and desiring to reduce the coefficients of less significant features to zero, ridge regression is a valuable tool. For example, in antenna design, where multiple input properties are considered, some of which may have no influence on the result [34].

**Bayesian Linear Regression:** Bayesian linear regression is used to determine the prior probability for the model parameters rather than to get the “optimal” value of the model parameters [35]. One benefit of using Bayesian Linear Regression is that the posterior distribution may be used to quantify the degree of uncertainty in the model's predictions. When the forecasts have to be understood probabilistically, this can be beneficial.

**Random Forest Regression:** To train various decision tree models, Random Forest Regression chooses random portions of the data. [36] Random Forest Regression is useful in antenna design because it can deal with high-dimensional data and non-linear connections between characteristics and the target variable.

**XGB Regression:** When training on massive datasets, XGBoost's built-in optimizations speed up the process. Regularization, parallel processing, and the ability to deal with missing values are just some of its cutting-edge features. Antenna designers can use simulated or measured data to make predictions about antenna properties like radiation patterns, gain, and directivity with the help of XGBoost [37].

**Gaussian Process Regression:** Gaussian process regression (GPR) is an example of a supervised machine learning technique that may be applied to regression and classification issues. GPR offers several advantages, such as the ability to perform successfully even with small datasets and to provide uncertainty metrics for predictions [38].

### 5.3 Performance metrics

Google provides a simulated Python environment called google colab, where researchers did all their machine learning research. The sci-kit learn machine learning framework was utilized to effectively build the Regression models. Matplotlib was used throughout, especially in the end, while analyzing and visualizing the results. As a result of its prevalence, error is the primary statistic in regression analysis. The concept of error is easily grasped. Various statistical indicators were used to assess the algorithms' efficacy, and those results were then compared against one another.

Six independent statistics—the mean absolute error (MAE), the mean squared error (MSE), the root mean square error (RMSE), the root mean squared logarithmic error (RMSLE), the coefficient of determination (R2), and the variance score—were used to evaluate the accuracy of the predictions.

Mean Absolute Error (MAE) quantifies the disparity between the predicted and actual values in a regression problem. High accuracy in predicting the dependent variable is indicative of a smaller MAE. Equation (1) depicts the MAE [39] formulation.

(1) $$MAE=\frac{1}{n}\sum_{i=1}^{n}\left|y_{i}-{\overset{ˆ}{y}}_{i}\right|$$

Where n is the total number of observations, ${\overset{ˆ}{y}}_{i}$ and $y_{i}$ represent the predicted and actual values.

Mean Squared Error (MSE) is a frequently employed metric for determining the effectiveness of a regression model. Because it can be quickly comprehended and analyzed, the MSE has gained widespread acceptance as a metric of choice. The MSE [40] formulation is shown in Equation (2).

(2) $$MSE=\frac{1}{n}\sum_{i=1}^{n}{\left(y_{i}-{\overset{ˆ}{y}}_{i}\right)}^{2}$$

The Root Mean Squared Error (RMSE) is a measure that is frequently utilized for the purpose of evaluating regression models. A smaller RMSE number indicates that the model is more accurate in its predictions. Equation (3) illustrates RMSE [41] expression.

(3) $$RMSE=\sqrt{\frac{1}{n}}\sum_{i=1}^{n}{\left(y_{i}-{\overset{ˆ}{y}}_{i}\right)}^{2}$$

Root Mean Squared Logarithmic Error (RMSLE) is a prediction error metric that computes the square root of the average logarithm of the squared differences between predicted and actual values in regression problems with a positive target variable. The equation of RMSLE [42] is shown in Equation (4).

(4) $$\text{ RMSLE }=\sqrt{(\log\left(y_{i}+1\right)-\log{\left({\overset{ˆ}{y}}_{i}+1\right)}^{2}}$$

The R-squared value indicates the accuracy of the model fit. When R2 is close to 1, it indicates that the model provides a good fit for the data, whereas when it is closer to 0, it indicates that the model is not all that good. When a model predicts an absurd outcome, R-squared can be negative. R-squared [43] is expressed in equation (5)

(5) $$R^{2}=1-\frac{\sum_{i=1}^{N}{\left(y_{i}-{\overset{ˆ}{y}}_{i}\right)}^{2}}{\sum_{i=1}^{N}{\left(y_{i}-{\overset{ˆ}{y}}_{i}\right)}^{2}}$$

The variance score is a statistical measure indicating how much of the overall variance in the dependent variable can be attributed to the model's independent variable(s). When the variance score is high, it means the model is a strong fit for the data. [44] describes the error dispersion in each dataset. It is defined as in equation (6)

(6) $$\text{ explained variance }(y,\overset{ˆ}{y})=1-\frac{\mathrm{Var}(y-\overset{ˆ}{y})}{\mathrm{Var}(y)}$$

### 5.4 ML based analysis

**Figure 17:**
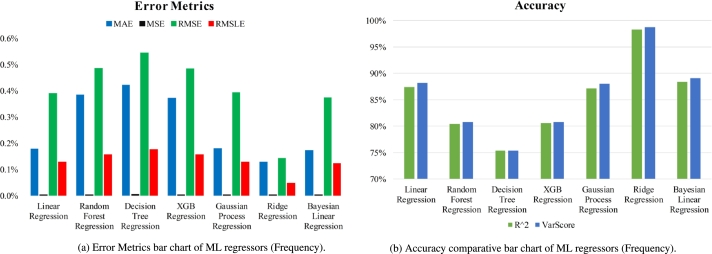
Performance comparative bar chart of ML regressors (Frequency).

Table 2 summarizes the results of a comparison between seven regression models' abilities to predict resonant frequency given six input parameters. The accuracy performance of each algorithm is measured using the MAE, MSE, RMSE, and RMSLE scores were 0.1269%, 0.0002%, 0.1414%, and 0.0455%, respectively. When it comes to R-squared and variance scores, Ridge Regression has the highest accuracy at 98.3275% and 98.7676%, respectively. Fig. 17(a) displays the error metrics bar chart whereas Fig. 17(b) depicts accuracy comparison results of different models.

**Table 2:** Performance metrics of ML regressors (Frequency).

Algorithms	MAE	MSE	RMSE	RMSLE	R^2^	Var Score
Linear Regression	0.1764%	0.0015%	0.3881%	0.1257%	87.4035%	88.2223%
Random Forest Regression	0.3821%	0.0023%	0.4842%	0.1553%	80.3905%	80.7910%
Decision Tree Regression	0.4207%	0.0029%	0.5423%	0.1740%	75.3993%	75.4101%
XGB Regression	0.3711%	0.0023%	0.4822%	0.1546%	80.5542%	80.7995%
Gaussian Process Regression	0.1788%	0.0015%	0.3919%	0.1270%	87.1536%	88.0020%
Ridge Regression	0.1270%	0.0002%	0.1414%	0.0455%	98.3276%	98.7677%
Bayesian Linear Regression	0.1712%	0.0014%	0.3727%	0.1208%	88.3786%	89.0847%

**Figure 18:**
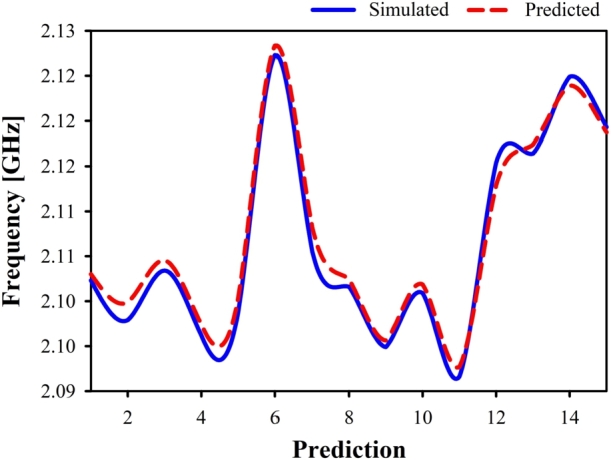
Simulated vs predicted frequency using Ridge Regression.

Fig. 18 displays the simulated and predicted results using ridge regression (RR) for 15 test samples. The simulated and predicted resonance frequencies and values are shown in Table 3. The bar chart of ML regression is separated into two sections. One is for error metrics (MAE/MSE/RMSE/RMSLE), while the other is for accuracy computation (R square/var score). In the study, we tune the frequency between 1.7 GHz and 2.7 GHz. Fig. 18 shows that this anticipated outcome is similar to the simulated one. Since RR is superior to alternative ML models in terms of frequency prediction, it is selected. It has been demonstrated that there is a marginal difference between the observed frequencies of RR and their predictions. The majority of the test samples, which consisted of a total of 15, were successfully predicted, with an error percentage of zero.

**Table 3:** Simulated and predicted resonant frequency comparison on the test set using Ridge Regression.

No.	Simulated Frequency	Predicted Frequency	Error	No.	Simulated Frequency	Predicted Frequency	Error
1	2.102300	2.103005	0.0336%	9	2.094900	2.095624	0.0346%
2	2.097900	2.099877	0.0942%	10	2.100900	2.101850	0.0452%
3	2.103400	2.104516	0.0531%	11	2.091600	2.092680	0.0516%
4	2.096200	2.097413	0.0579%	12	2.115400	2.112861	0.1200%
5	2.098700	2.100312	0.0768%	13	2.116400	2.117401	0.0473%
6	2.127300	2.128354	0.0495%	14	2.124900	2.123924	0.0459%
7	2.105500	2.108161	0.1264%	15	2.119300	2.118730	0.0269%
8	2.101600	2.102471	0.0415%				

**Figure 19:**
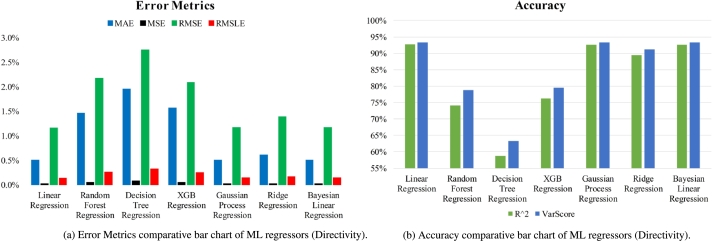
Performance comparative bar chart of ML regressors (Directivity).

For directivity, in terms of the most excellent R square and variance scores, respectively 92.7136% and 93.337%, the Linear Regression model performs better than the test set, according to Table 4. In terms of percentage, the values for MAE were 0.5005%, MSE were 0.0132%, RMSE were 1.1513%, and RMSLE were 0.1341%. The Gaussian Process Regression came in second for predicting the directivity. It has the variance score and the second-highest R2 score. Additionally, the second-last possible marks for MAE, MSE, RMSE, and RMSLE. In addition, a bar graph is used to compare the results of different models in Fig. 19. The bar chart representing the ML regression may be broken down into two distinct pieces. One is for computing error measures such as MAE, MSE, RMSE, and RMSLE depicted in Fig. 19(a), while the other is for computing accuracy using R square and var score shown in Fig. 19(b).

**Table 4:** Performance metrics of ML regressors (Directivity).

Algorithms	MAE	MSE	RMSE	RMSLE	R^2^	Var Score
Linear Regression	0.5005%	0.0133%	1.1514%	0.1342%	92.7137%	93.3371%
Random Forest Regression	1.4599%	0.0471%	2.1697%	0.2512%	74.1253%	78.8163%
Decision Tree Regression	1.9435%	0.0751%	2.7407%	0.3176%	58.7130%	63.2233%
XGB Regression	1.5625%	0.0433%	2.0806%	0.2411%	76.2064%	79.4493%
Gaussian Process Regression	0.5022%	0.0134%	1.1580%	0.1349%	92.6289%	93.2907%
Ridge Regression	0.6018%	0.0191%	1.3825%	0.1610%	89.4944%	91.1889%
Bayesian Linear Regression	0.4998%	0.0134%	1.1580%	0.1349%	92.6288%	93.2861%

**Figure 20:**
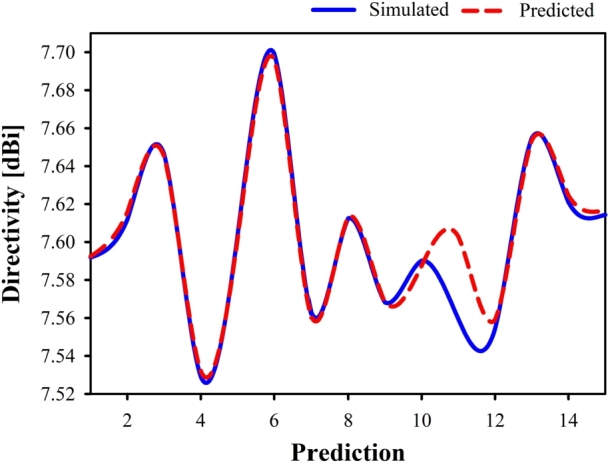
Simulated vs predicted directivity using Linear Regression.

Table 5 displays the relative difference between the simulated and predicted directivities. The test set for this study consists of fifteen samples, which account for 20% of the whole dataset. Fig. 20 compares the simulated directivity to the predicted directivity using linear regression. It has been demonstrated that the actual and expected directivities for LR differ from one another by a tiny amount. The majority of the 15 test samples could be predicted with a high degree of accuracy, with an error percentage that was very close to zero.

**Table 5:** Simulated and predicted directivity comparison on the test set using Linear Regression.

No.	Simulated Directivity	Predicted Directivity	Error	No.	Simulated Directivity	Predicted Directivity	Error
1	7.591829	7.592457	0.0083%	9	7.568187	7.568415	0.0030%
2	7.611878	7.615796	0.0515%	10	7.590142	7.587388	0.0363%
3	7.646371	7.645021	0.0176%	11	7.559451	7.602974	0.5757%
4	7.529098	7.532055	0.0393%	12	7.554280	7.559007	0.0626%
5	7.603126	7.601331	0.0236%	13	7.654765	7.653481	0.0168%
6	7.699371	7.696373	0.0389%	14	7.620946	7.624627	0.0483%
7	7.562601	7.560499	0.0278%	15	7.614383	7.616704	0.0305%
8	7.612544	7.613361	0.0107%

## 6 Conclusions

This study describes a quasi-Yagi antenna for the UMTS 2100 MHz band of the LTE communication system and then forecasts its directivity and frequency using a number of machine-learning approaches. The initial design of a quasi-Yagi antenna using CST yields encouraging results in a low return loss of -38.40 dB, a maximum gain of 6.9 dB, and a maximum directivity of 7.67 dBi. The antenna also has a VSWR of 1.001 at the center frequency and a maximum efficiency of 89.9%. The results of CST have also been validated by developing an equivalent RLC circuit model and simulating it in the ADS. Both the CST and ADS simulators provide reflection coefficients that are pretty comparable to one another. Seven different machine-learning techniques were finally put into practice. The ridge regression model outperforms the test data in terms of forecasting resonant frequencies. However, the linear regression (LR) model performs better than other models when it comes to forecasting directivity. On the other hand, we were constrained by computational limitations, which led us to collect a total of 75 samples for the purpose of machine learning modeling. Consequently, we were unable to incorporate deep learning models into our experiment. Additionally, the suggested prototype has been created and put through testing in the lab. The designed Yagi antenna offers UMTS frequency bands, and there is excellent consistency between modeling and experimental findings. Though the proposed antenna volume is somewhat large $\left(75\times 100\times 1.6mm^{3}\right)$, it uses a very low-cost dielectric substrate, FR4. It is clear from analyzing these factors that the suggested antenna is a great choice for the UMTS 2100 MHz LTE communication system.

## Funding statement

This research did not receive any specific grant from funding agencies in the public, commercial, or not-for-profit sectors.

## CRediT authorship contribution statement

Md. Ashraful Haque, Dipon Saha, Samir Salem Al-Bawri, Liton Chandra Paul, Md Afzalur Rahman, Faisal Alshanketi, Ali Alhazmi, Ali Hanafiah Rambe, M.A. Zakariya & Saeed S. Ba Hashwan: Conceived and designed the experiments; Performed the experiments; Analyzed and interpreted the data; Contributed reagents, materials, analysis tools or data; Wrote the paper.

## Declaration of Competing Interest

The authors declare no conflict of interest.

## Data Availability

No data was used for the research described in the article.
